# Ultra-Light Clay Intervention Improves Responsiveness and Initiates the Communication of Children With ASD

**DOI:** 10.3389/fpsyg.2022.804488

**Published:** 2022-03-14

**Authors:** Jing Zhang, Qingzhou Sun, Xue Liu, Fuyi Yang

**Affiliations:** ^1^Department of Special Education, Faculty of Education, East China Normal University, Shanghai, China; ^2^School of Management, Zhejiang University of Technology, Hangzhou, China; ^3^School of Environmental and Natural Resources, Zhejiang University of Science and Technology, Hangzhou, China

**Keywords:** children with ASD, ultra-light clay intervention, responsive to communication, initiation of communication, peer-generalization effect

## Abstract

The barriers to responsiveness and the initiation of communication are the two key problems encountered by children with autism spectrum disorders (ASD). Prior interventions based on behavioral reinforcement have had an obvious effect on responsive communication but a weak effect on the initiation of communication. Based on psychological development theory, we designed ultra-light clay interventions involving hands-on production or multi-interaction around key concepts and themes, teaching children about basic concepts, relationships, and logic, making abstract knowledge concrete and experience. Two studies (Study 1: *N* = 3, one-to-one intervention; Study 2: *N* = 8, one-to-two intervention) showed that ultra-light clay intervention improved both the initiation of and response to communication among children with ASD, but that such improvements show a peer-generalization effect in initiation communication, not in responsive communication. These findings provide a set of ultra-light clay interventions for communication in children with ASD and suggest a relationship between endogenous interventions and the initiation of communication.

## Introduction

Autism spectrum disorders (ASD) are characterized by impairments in social interaction and communication, together with restricted, repetitive, and stereotyped patterns of behavior, interests, and activities (American Psychiatric Association, 2013). Statistics from the Autism and Developmental Disabilities Monitoring (ADDM) Network of the Federal Centers for Disease Control and Prevention (CDC) show that the number of children with ASD is increasing (e.g., the ratio increased from 1/60 in 2014 to 1/54 in 2016; Knopf, 2020). In China, more than 10 million people are autistic, including 2 million children aged 0–14 years (China net., 2019). Children with ASD face many social problems, and in many cases, their families suffer a great deal of pain and burden. The key problem that these children encounter is a barrier to communication (Pickles et al., 2009; Koegel and Koegel, 2018; Hampton et al., 2020), including decreased academic performance, increased problematic behavior, and difficulties forming relationships with others and integrating into a typical society (Koegel et al., 1992; Cholewicki et al., 2019).

Communication is the basis of interaction with people, and it includes two levels of meaning: responsive communication and initiating communication. However, the lack of responsiveness and difficulty in initiating communication are the two major communication barriers of children with ASD (Mundy et al., 1990; Siller and Sigman, 2002). Responsive communication reflects the verbal response to others’ words or behaviors (McGee and Daly, 2007), and it often begins in the early stages of language development (Kristelle et al., 2010). Initiating communication is the ability to use language to make a request or express a thought or feeling (Kasari et al., 2008). It reflects children’s understanding, internalization, and experience of situations (National Research Council, 2001). Initiating communication often starts in the advanced stages of children’s language development (Fenson et al., 1994); thus, it requires more cognitive and emotional participation than responsive communication.

The communication abilities of children with autism vary greatly, and intervention research has generally not used a between-group design but a single-subject design (Koegel et al., 1997). Previous communication interventions for children with ASD were based on a technique of behavior modification known as applied behavior analysis (Hume et al., 2014). Specifically, some studies adopted stimulus–response reinforcement, which has a remarkable effect on responsiveness but not on initiation (Ben-Itzchak and Zachor, 2007; Lancioni et al., 2007). For example, Lovaas (1987) used the method of saying “yes” or clapping for appropriate behaviors in children with ASD and saying “no” for their inappropriate behavior and found that these reinforcements improved children’s ability to respond verbally. Lancioni et al. (2007) adopted stimulus–response reinforcement to establish the connection between contextual images and verbal content and observed an obvious improvement in the responsive communication of children with ASD. Ben-Itzchak and Zachor (2007) observed that behavioral reinforcement improved the verbal response of children with ASD (e.g., responding to joy or anger), though it did not improve children’s ability to initiate communication (e.g., expressing joy or anger proactively). Similarly, Hampton et al. (2020) also revealed that behavioral reinforcement (e.g., prompt reinforcement or “ask-answer” reinforcement) promoted language imitation and response in children with ASD but did not improve children’s initiation in the changed context. This is not surprising given that initiating is considered a pivotal behavior and an essential skill in social communication (Koegel and Koegel, 2018). Koegel et al. (2001), Koegel and Koegel (2006), and Koegel and Koegel (2018) assert that initiating communication leads to an array of additional social gains because it provides additional verbal and social learning opportunities. Therefore, it is a commonly targeted behavior in social skills interventions for children with ASD.

Researchers in language development offer explanations for this. Compared to responsive communication, initiating communication requires a higher level of cognitive and emotional involvement (Schertz and Odom, 2006; Koegel and Koegel, 2018). Responsive communication involves “stimulus” and “response,” and this exogenous intervention, by strengthening the connection between “stimulus” and “response,” is effective for improving responsive communication in children with ASD (Jones, 2009). However, initiating communication involves more complex processes, such as “understanding,” “internalization,” “coding,” “extracting,” “output,” and so on (Kristelle et al., 2010). The initiation of communication is based on an individual’s language internalization, such as requirement analysis, language transformation, and language output (Koegel and Koegel, 2018; Hampton et al., 2020). Behavioral interventions by stimulus–response reinforcement do little to promote language understanding and internalization and thus hardly promote the initiation of communication among children with ASD. From the perspective of children’s language development, initiating communication often lags behind responsive communication (Leekam et al., 2000). The latter is mainly driven by exogenous stimuli, while the former mainly by endogenous motivation. In summary, endogenous intervention may be effective for improving the ability to initiate communication in children with ASD. Vygotsky’s psychological development theory provides further insights into language development.

Vygotsky et al. (1979) argue that children’s language development is driven by two aspects: exogenous and endogenous drives. The former reflects an individual’s response to external “stimuli,” while the latter reflects an individual’s understanding, internalization of external knowledge, and proactive output of internal experience (Preissler, 2006). Endogenous drive involves the concretization of abstract knowledge, vividness of mundane knowledge, interest in receiving knowledge, and experience of knowledge rather than the description of it. Based on this theory, a communication intervention that could make abstract knowledge more concrete, vivid, interesting, and experienced (focusing on endogenous drive) should improve the ability to initiate communication in children with ASD. The emergence of ultra-light clay has provided inspiration for this.

Ultra-light clay intervention refers to using hands-on production or multi-interaction around key concepts and themes with the medium of ultra-light clay to teach children basic concepts, relationships, and logic, making abstract knowledge concrete, vivid, interesting, and experienced (Rahmani and Moheb, 2010). Ultra-light clay intervention based on psychological development theory is conducted in a natural situation, and both the physical environment and the activity situation can be embedded in daily life. Therefore, the systemicity of the intervention is emphasized, especially for various age-appropriate activity materials, which will be an effective tool for communication (Hume et al., 2014). Ultra-light clay intervention has three main advantages: (1) based on applied behavior analysis, it contains rich color stimuli, while numerous studies have highlighted the value of visual strategies in children with autism (Tissot and Evans, 2003; Hume et al., 2014); therefore, children’s learning strategies can be continuously improved by providing visual support, (2) making abstract concepts more concrete and easier to understand through visual cues (Koegel et al., 2015, 2016). Similar to plastic modeling, ultra-light clay can not only arouse the interest of children with ASD, but it can also present abstract concepts in a concrete form (Schaefer and Kaduson, 2006). For example, the interventionist can input abstract concepts (e.g., knead, pinch, rub, big, small, light, and heavy) into concrete, vivid, and experienced operations, thereby improving the concepts of internalization and understanding (Rahmani and Moheb, 2010). We speculate that ultra-light clay intervention can improve the ability to initiate communication because of its endogenous drive, and (3) application prospect. Ultra-light clay is easy to manipulate, is adjustable in terms of difficulty (e.g., the therapist can set tasks with different difficulties), and is diversified in intervention forms (e.g., one-to-one form: one interventionist working with one child with ASD; one-to-N: one interventionist working with two or more children with ASD). The intervention is suitable for children with different degrees of ASD and of various ages. For instance, career-oriented activities, such as pastry making or clay sculpture, can be designed for older children or for those with a mild degree of autism, and hand-eye coordination activities, such as sticking or pressing the clay, can be designed for younger children or those with more severe autism (Kaduson and Schaefer, 1998). In addition, the one-to-one form is better for tracking children’s verbal changes, while the one-to-N form is more suitable for peer communication and cooperation. The multi-interaction around the key themes should promote peer communication and generate a peer generalization effect. Therefore, if the effect of ultra-light clay intervention is effective for communication barriers in children with ASD, it should have potential for great applications. One study using ultra-light clay intervention in children without autism provided support for our hypothesis: Sherwood (2004) adopted ultra-light clay intervention and obtained good results in improving children’s cognitive, emotional, and verbal development. However, it is still unknown whether it can improve the responsive and initiating communication of children with ASD.

In the current research, we designed a series of interventions based on ultra-light clay to explore the effects on the ability to respond to and initiate communication in children with ASD. To explore the effect of the intervention on individual behavioral improvement, we used a single-subject experimental design to verify the effectiveness of the intervention, which emphasizes measures of behavioral change in only one or a few participants. Due to the strong individual differences in children with autism, each individual has their own uniqueness; therefore, the single-subject design is the most suitable intervention method and has high ecological validity. In Study 1, we adopted the one-to-one form and used an ABABA design to examine whether ultra-light clay interventions would improve the responsive and initiating communication of children with ASD. In Study 2, we adopted the one-to-N form and used an ABA design to further test the effect of ultra-light clay intervention and to explore whether a peer generalization effect exists.

## Study 1

### Subjects and Design

In this study, we adopted the one-to-one intervention form, a single-subject ABABA design, to examine the effect of ultra-light clay interventions on responsive and initiating communication in children with ASD.

Considering the intervention period and the difficulty of the study, we recruited three children with autism (range from 8.83 to 9.50 years, M = 9.08, SD = 0.34) from a special education school in Wuxi, China (Table 1) after referring to the research of Dionne and Martini (2011) and Tan and Alant (2016) and so on. The inclusion criteria for the participants were as follows: (1) had been previously diagnosed by two experienced pediatric psychiatrists; (2) met the diagnostic criteria for autism based on the Diagnostic and Statistical Manual of Mental Disorders (American Psychiatric Association, 2013); (3) since the Autism Diagnostic Observation Schedule (ADOS, Lord et al., 2000) has not been widely adopted for use in China, we used the Chinese version of the Childhood Autism Rating Scale (CARS) developed by Schopler et al. (1988) to confirm the diagnosis, and the CARS scores of the participants were all above the cut-off score of 30; the higher the score, the heavier the degree; (4) participants’ communication levels were matched by interviews with their major teachers and primary caregivers. Teacher interviews were administered at school, and parent interviews were administered either at home or at school. The data were collected at baseline, prior to randomization and delivery of any trial intervention; and (5) the students had received their guardians’, schools’, and the Ethics Committee’s permission to participate in the study.

**Table 1:** The participants’ details (Study 1).

	ASD_1_^1^	ASD_2_	ASD_3_	*M* (±SD)
Age (year, month)	8,10	9,1	9,6	9,1 (0.34)
Gender	Male	Male	Female	
CARS	31	36	48	47.00 (9.64)

1 The abbreviation of the name, to protect the participant’s privacy.

The exclusion criteria for the participants were as follows: (1) history of head trauma, (2) neurological or mental disorder, and (3) neurological or psychiatric drugs.

### Intervention Process

We conducted the intervention in a familiar, quiet classroom for children with autism. The classroom had only one table, two chairs, and prepared ultra-light clay material to eliminate extra distractions. The interventionist and child sat side by side. The intervention lasted for 6 months, three times a week, and for 25–30 min each time. The specific process was as follows: (1) evaluation stage: we used the adapted ecological evaluation scale (Brown et al., 1979) to assess the state (e.g., advantages, cognitive abilities, emotional abilities, etc.) and growing environment (e.g., family, school, community, etc.) of these children. We then conducted an appropriate intervention plan for each child. (2) Baseline (A_1_): we observed and recorded their communication behaviors—both responsive and initiated—in a natural state without interventions (six times). (3) Intervention (B_1_): implementing ultra-light clay interventions. We observed and recorded their responsive and initiating communication behaviors during these interventions (10 times). (4) Baseline (A_2_): stopping interventions. We observed and recorded their responsive and initiating communication behaviors in a natural state (six times). (5) Intervention (B_2_): Resuming interventions. We recorded their responses and initiating communication behaviors (21 times). (6) Baseline (A_3_): Stop interventions again. We recorded their responses and initiating communication behaviors in a natural state (six times). Interventions were recorded using a SONY DV camera.

### Intervention Content

Based on previous intervention methods (e.g., Kaduson and Schaefer, 1998; Rahmani and Moheb, 2010), we set up the following specific content: kneading exercises, making a single item, making a series of themes, making large themes, making imaginary items, and creating imaginary themes (Table 2).

**Table 2:** Intervention contents and interventionists’ strategies (Study 1).

Control	Process	Aims	Achievements	Interventionists’ strategies
Kneading exercises	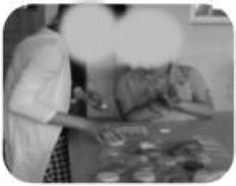	Familiar with materials understand basic techniques	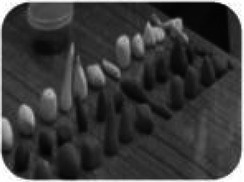	Ask questions, identify
Basic perception	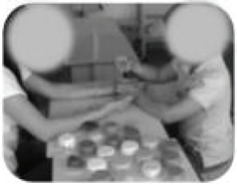	Familiar with basic colors and shapes	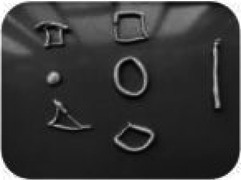	Find the same color, color matching, mold perception scribe, knead
Single item	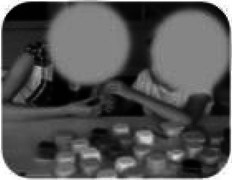	Understand the composition and characteristics of the item	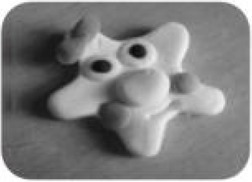	Demonstrate imitate, wait
Series of themes	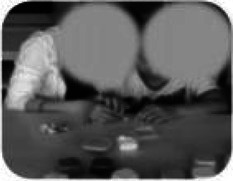	Understand the relationships between related objects	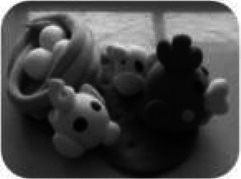	Role play, social stories extend to personal experiences, basic social etiquette, etc.
Large theme	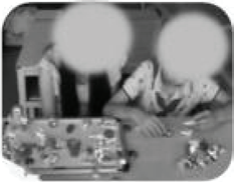	Develop subject’s imagination in the form of stories	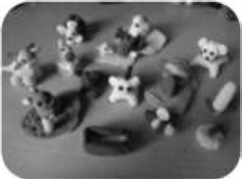	Guide the production of items related to the theme in the form of stories
Imaginary item	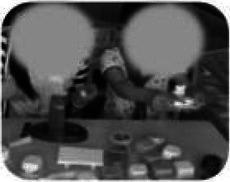	Incorporate life experiences in activities	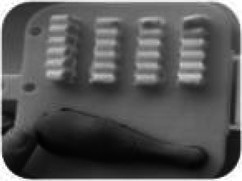	Integrate life experience with interests, develop individual imagination, and guide active expression
Imaginary theme	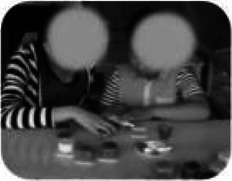	Develop imaginative topics that are interest-based	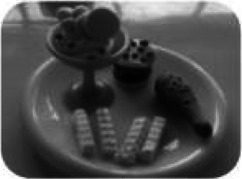	Unfold the production of imaginative themes with stories designed by individuals

**Kneading exercises:** Becoming familiar with the materials and understanding the basic techniques (kneading, rubbing, pressing) and concepts (size, number, weight) through questions and identification.

**Making single items:** By making single items, the subjects could understand the proportions of clay, color matching, and flow chart. Our items included star, octopus, cat, dog, pigs, hedgehog, lion, carrot, pea, corn, and watermelon.

**Making series of themes:** Creating objects composed of multiple images, using strategies such as role play, social stories, or other strategies. The subjects were guided to understand the relationship between relevant objects and to learn everyday names for objects (e.g., chicken mother and baby) as well as basic etiquette (e.g., hello chicken mother, I am chicken baby). The themes were hen laying eggs, goose drinking water, rabbit eating carrots, duck swimming, and so on.

**Making large themes:** Combining single objects with a series of themes into a big “farm” theme, training individual imagination in the form of stories (e.g., farm animals are thirsty, so I need to make a sink), finding steps online, and making items independently. Items that the subjects made included a sink, a television, a tractor, and so on.

**Making imaginary items:** Integrate life experience into the intervention and guide the subjects to actively express the items they wanted to make (drumstick, hamburger, French fries, etc.).

**Making imaginary themes:** Combining imaginary items into a “food” theme and matching the imaginary theme (e.g., ice cream, cake) to the subjects’ interests.

### Inter-Observer Agreement

**Responsive communication index:** we recorded the total number of communications initiated by the researcher and the number of effective responses from the subject in each activity. The ratio (the number of effective responses from the subject to the total number of communications initiated by the researcher) reflects the percentage of the subject’s effective responsive communication; the higher the value, the higher the responsive communication behavior.


$$\begin{array}{l} \mathrm{Index ofresponsive communication}=\\ \frac{\mathrm{the}\;\mathrm{number}\;\mathrm{of}\;\mathrm{effective}\;\mathrm{responsives}\;\mathrm{from}\;\mathrm{subject}}{\mathrm{the}\;\mathrm{total}\;\mathrm{number}\;\mathrm{of}\;\mathrm{communications}\;\mathrm{initiated}\mathrm{by}\mathrm{the researcher}} \end{array}$$

**Initiating communication index:** we recorded the total number of communications initiated by the subject and the time of each intervention. The ratio (the total number of communications initiated by the subject/the time of each intervention) reflects the subject’s initiating communication per unit of time; the higher the value, the higher the initiating communication behavior.


$$\begin{array}{l} \mathrm{Index of initiatingcommunication}=\\ \frac{\mathrm{the}\;\mathrm{total}\;\mathrm{number}\;\mathrm{of}\;\mathrm{communications}\;\mathrm{initiated}\mathrm{by}\mathrm{the researcher}}{\mathrm{the time of each intervention}} \end{array}$$

Before the study, observers were trained by coding sample videos using the coding standards until each coder reached a minimum 85% agreement. The observers were one teacher, one graduate student, and the researcher. Inter-observer agreement (IOA) was calculated by dividing the total agreement by the sum of agreements and disagreements and multiplying by 100 to convert the result into a percentage. For all subjects, the IOA across phases was 97% (range = 92–100%) for responsive communication and 94% (range = 92–100%) for initiating communication.

### Procedural Fidelity

The interventionist works with children, playing the role of question-asking and guiding. Procedural fidelity was measured using a checklist for quality components for each condition and for rates of strategy use, as captured in the coded transcripts.

For baseline phrases, the checklist consisted of the following steps initiated by the researcher: (1) read the list of tasks for this class; (2) open the box and take out the clay; (3) affirm the children’s operation and vocally describe it by saying, “Yes, this is kneading the clay;” (4) use an attention-getting word (e.g., “Look,” “Wow”) while demonstrating the steps; (5) wait 5 s while looking expectantly at the child; (6) implement a step-by-step process according to the intervention content and strategies (see Table 2); (7) check the list of tasks and tick off items; (8) put the clay back in the box; and (9) repeat steps b–h for the remaining items in the task.

For the intervention sessions, the checklist consisted of the following steps initiated by the researcher: (1) read the list of tasks for this class; (2) open the box and have the children take turns removing the clay (e.g., “It is your turn”) and wait 5 s while looking expectantly at the child; (3) affirm the operation of the children and vocally describe it; (4) praise the child by providing specific natural feedback; for example, when the child presses clay, describe it by saying, “Yes, this is pressing the clay;” (5) use an attention-getting word (e.g., “Look,” “Wow”) while demonstrating the steps; (6) implement a step-by-step process according to the intervention content and strategies (see Table 2); (7) check the list of tasks and tick off items; (8) put the clay back in the box; and (9) repeat steps (2)–(8) for the remaining items in the task.

The percentage of correct implementation was calculated by dividing the total number of steps performed correctly by the total number of checklist steps and multiplying by 100. The scores remained high across study conditions with minimal variability, with an average of 100% during baseline and 89% (range = 83–100%) during the intervention.

### Results

We conducted two one-way ANOVA analyses of the subjects’ responsive communication and initiating communication as the dependent variables and five intervention stages as independent variables.

#### Responsive Communication

**Figure 1:**
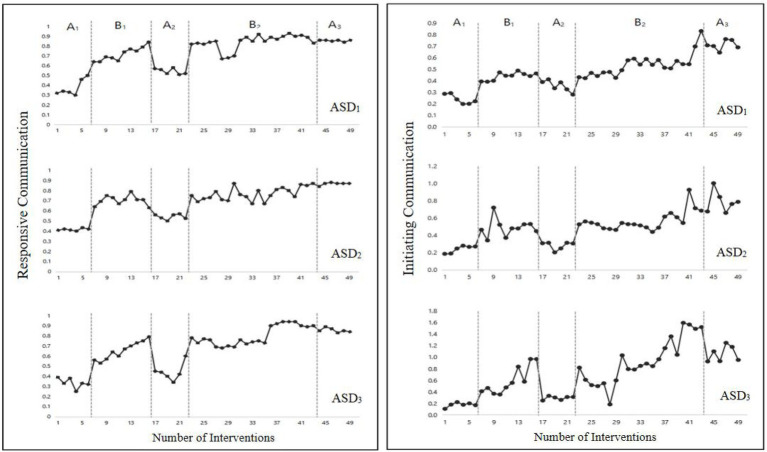
Changes of responsive (left side) and initiating (right side) communication in the five intervention stages (Study 1).

A significant difference was found in responsive communication among the five stages (A_1_, B_1_, A_2_, B_2_, A_3_), *F*(4,44) = 112.66, *p* < 0.001 (Figure 1-left side). In particular, the response communication in the first intervention stage (B_1_) was significantly higher than that in the first baseline stage (A_1_), *M_B1_* = 0.69, *M_A1_* = 0.37, *F*(1,14) = 161.41, *p* < 0.001. This indicates that the subjects’ responsive communication improved after the intervention. Responsive communication in the second baseline stage (A_2_) was significantly lower than in the first intervention stage (B_1_), *M_A2_* = 0.51, *F*(1,14) = 56.20, *p* < 0.001. This indicates that the subjects’ responsive communication decreased after the withdrawal of the intervention, which suggests a preliminary confirmation of the intervention’s effectiveness. Responsive communication in the second intervention stage (B_2_) was significantly higher than in the second baseline stage (A_2_), *M_B2_* = 0.80, *F*(1, 25) = 116.30, *p* < 0.001. This indicates that the subjects’ responsive communication improved again after we reimplemented the intervention, thus eliminating the interference of the subjects’ own growth. Responsive communication in the third baseline stage (A_3_) was significantly higher than in the second intervention stage (B_2_), *M_A3_* = 0.86, *F*(1, 25) = 4.13, *p* < 0.05. This showed that the subjects’ performance in responsive communication remained good after the intervention’s withdrawal.

#### Initiating Communication

A significant difference was found in initiating communication among the five intervention stages (A_1_, B_1_, A_2_, B_2_, A_3_), *F*(4, 44) = 28.09, *p* < 0.001 (Figure 1-right side). Initiating communication in the first intervention stage (B_1_) was significantly higher than in the first baseline stage (A_1_), *M_B1_* = 0.51, *M_A1_* = 0.22, *F*(1, 14) = 59.80, *p* < 0.001, which indicates that the participants’ initiating communication improved after the intervention. Initiating communication in the second baseline stage (A_2_) was significantly lower than in the first intervention stage (B_1_), *M_A2_* = 0.31, *F*(1, 14) = 27.47, *p* < 0.001, which indicates that after the intervention’s withdrawal, the subjects’ initiating communication decreased, which suggests a preliminary confirmation of the intervention’s effectiveness. Initiating communication in the second intervention stage (B_2_) was significantly higher than in the second baseline stage (A_2_), *M_B2_* = 0.68, *F*(1, 25) = 24.27, *p* < 0.001, which indicates that after the intervention was reimplemented, the subjects’ initiating communication improved again, eliminating the effect of the subjects’ own growth. Initiating communication in the third baseline stage (A_3_) was significantly higher than in the second intervention stage (B_2_), *M_A3_* = 0.85, *F*(1, 25) = 5.26, *p* < 0.05, indicating that the subjects’ performance in initiating communication was maintained well after the intervention’s withdrawal.

Study 1 shows that ultra-light clay intervention improves not only the subjects’ responsive communication but also their initiating communication. In Study 2, we adopted the one-to-N form to further test the effect of ultra-light clay intervention and to explore whether subjects’ responsive and initiating communication can be improved by communication with their peers.

## Study 2

### Subjects and Design

In this study, we adopted a one-to-two intervention form, a single-subject ABA design, to further test the effect of ultra-light clay intervention and to explore whether the responsive and initiating communication of children with ASD can be improved through peer generalization. This study is a further expansion of Study 1. The difficulty of the study is reduced and the time span is relatively short (ABA), so we increase the number of subjects.

We recruited eight children with autism^1^ (age ranging from 7.67 to 16.25 years, *M* = 10.06, SD = 3.36) from a special education school in Shenzhen, China (Table 3), they were divided into four pairs from grade 1 (*M* = 7.88), 2 (*M* = 9), 3 (*M* = 10.13), and 9 (*M* = 16). The inclusion and exclusion criteria were the same as those in Study 1. Each pair’s communication level was matched by interviews with their major teachers and primary caregivers. Teacher interviews were administered at school, and parent interviews were administered either at home or at school. Data were collected at baseline, prior to randomization and delivery of any trial intervention.

**Table 3:** The participants’ details (Study 2).

	ASD_4_^1^	ASD_5_	ASD_6_	ASD_7_	ASD_8_	ASD_9_	ASD_10_	ASD_11_	*M* (±SD)
Age	7,8	8,1	8,10	9,2	10,1	10,2	15,9	16,3	10,75 (3.36)
Gender	Male	Male	Male	Male	Male	Male	Male	Male	
CARS^4^	33	35	34	36	34	34	35	36	34.63 (1.06)

1 The abbreviation of the name, to protect the participant’s privacy.

### Intervention Process

We conducted intervention training for 3 months, twice a week, for 25–30 min each time. The specific process was as follows: (1) evaluation stage: same as in Study 1. (2) Baseline (A_1_): observing and recording participants’ responsive and initiating communication behaviors in a natural state without interventions (four times). (3) Intervention (B_1_): implementing ultra-light clay interventions and recording participants’ responsive and initiating communication behaviors during these interventions (10 times). (4) Baseline (A_2_): stopping interventions, recording their responses, and initiating communication behaviors in a natural state (four times). The interventions were recorded with a SONY DV camera.

### Intervention Content

**Figure 2:**
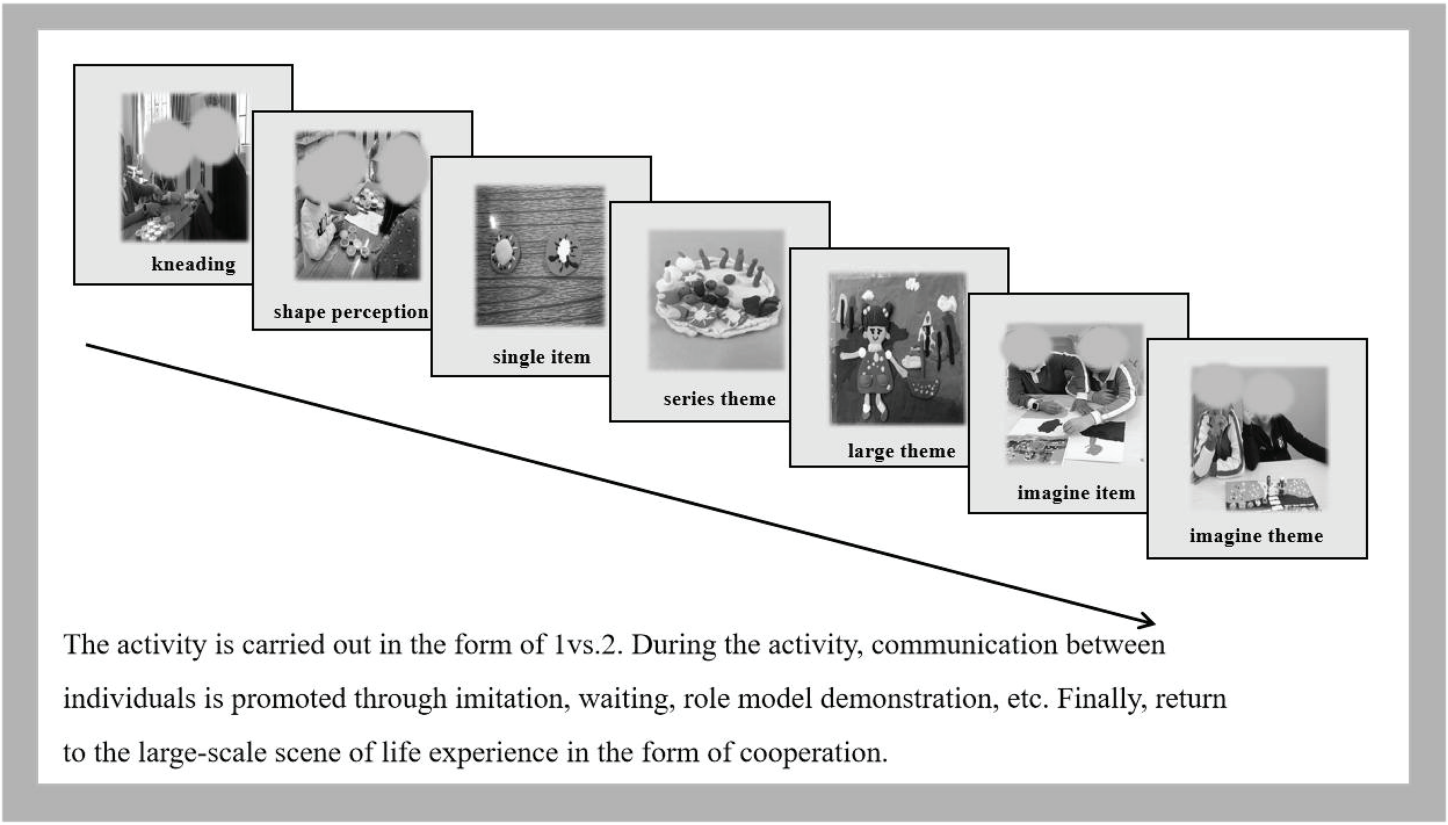
Intervention process (Study 2).

The intervention content is shown in Figure 2. Kneading exercises, making single items, creating a series of themes, creating large themes, making imaginary items, and creating imaginary themes were the same as in Study 1.

### Index Calculation

The responsive communication index and initiating communication index were the same as in Study 1, with the difference being that we recorded both the indexes for subject-interventionist and between-subject responsive communication and initiating communication in Study 2. The higher the values of the between-subject indexes, the higher the peer generalization effect.

### Inter-Observer Agreement and Procedural Fidelity

Same as in Study 1. For all subjects, the IOA across phases was 94% (range = 92–100%) for responsive communication and 96% (range = 90–100%) for initiating communication. The IOA for subject-subject frequency of responsive communication was 100 and 98% (83–100%) for initiating communication. The mean percentage of steps implemented correctly was 97% (90–100%) during baseline and 92% (range = 89–100%) during the intervention.

### Results

We conducted four one-way ANOVA analyses on subject-interventionist responsive communication, subject-subject responsive communication, subject-interventionist initiating communication, and subject-subject initiating communication as dependent variables and intervention stages as independent variables.

**Figure 3:**
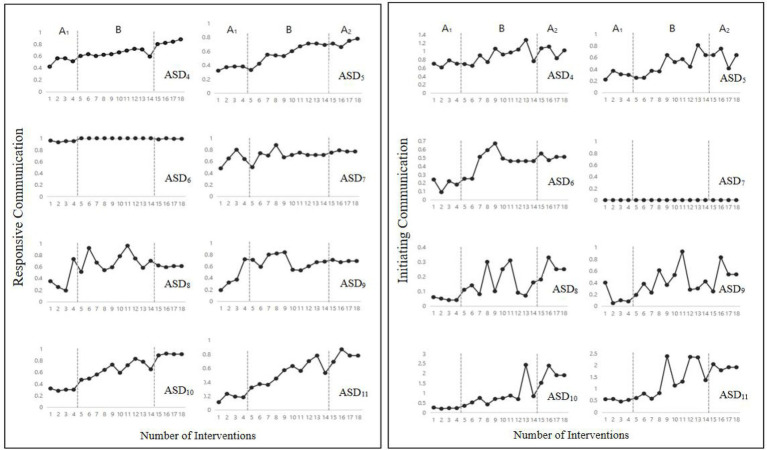
The change in subject-researcher responsive (left side) and initiating (right side) communication in the three intervention stages (Study 2).

Responsive Communication: (1) For subject-interventionist responsive communication, a significant difference was found among the three stages (A_1_, B, A_2_), *F*(2,141) = 26.46, *p* < 0.001 (Figure 3-left side); in particular, responsive communication in the first intervention stage (B) was significantly higher than that in the first baseline stage (A_1_), *M_B_* = 0.69, *M_A1_* = 0.47, *F*(1,110) = 28.06, *p* < 0.001. This indicates that the subjects’ responsive communication improved after the intervention. The responsive communication in the second baseline stage (A_2_) was higher than that in the first intervention stage (B), *M_A2_* = 0.79, *F*(1,110) = 9.73, *p* < 0.05, indicating that the subjects’ responsive communication was maintained well after the intervention’s withdrawal. (2) For the subject-subject responsive communication, no difference was found among the three stages (A_1_, B, A_2_), *F*(2,55) = 0.03, *p > 0*.05, which indicates that the improvement in the responsive communication of participants with ASD was not influenced by peer generalization.

**Figure 4:**
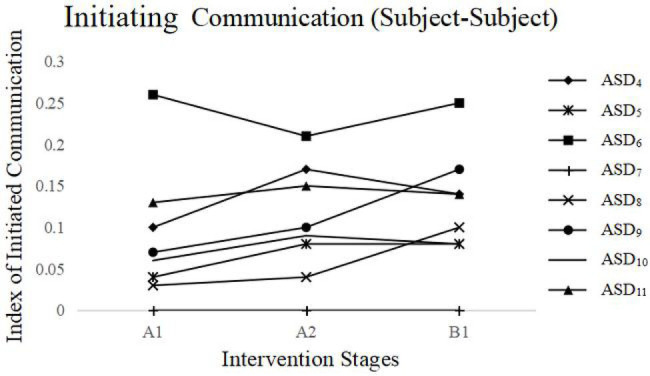
Changes in subject-subject initiating communication in the three intervention stages (Study 2).

Initiating Communication: (1) for the subject-interventionist initiating communication, a significant difference was found among the three stages (A_1_, B, A_2_), *F*(2,141) = 9.56, *p* < 0.001 (Figure 3-right side). Initiating communication in the first intervention stage (B) was significantly higher than in the first baseline stage (A_1_), *M_B_* = 0.58, *M_A1_* = 0.27, *F*(1,110) = 28.06, *p* < 0.001, and initiating communication in the second baseline stage (A_2_) was higher than in the first intervention stage (B), *M_A2_* = 0.84, *F*(1,110) = 4.75, *p* < 0.05. (2) For the subject-subject initiating communication, a significant difference was found among the three stages between subjects (A_1_, B, A_2_), *F*(2,55) = 3.74, *p* < 0.05. Initiating communication in the first intervention stage (B) was significantly higher than in the first baseline stage (A_1_), *M_B_* = 0.21, *M_A1_* = 0.17, *F*(1,46) = 4.49, *p* < 0.05, while it showed no difference in the second baseline stage (A_2_) and in the first intervention stage (B), *M_A2_* = 0.23, *F*(1,43) = 1.11, *p* > 0.05. This indicates that the subjects’ initiating communication was maintained well after the intervention’s withdrawal and showed good generalization between peers. It is worth noting that some individual differences existed between the subject-interventionist and subject-subject interventions (see Figures 3, 4).

Consistent with Study 1, Study 2 also showed a positive effect of ultra-light clay intervention on the responsive and initiating communication of children with ASD; in addition, it revealed a generalization effect between subjects in improving children’s initiating communication but not in responsive communication.

## Discussion

The current study explored how ultra-light clay intervention influenced the responsive and initiating communication behaviors of children with ASD. Two studies revealed that ultra-light clay interventions improved children’s responsive and initiating communications. In addition, such interventions showed a generalization effect between peers in initiating communication and not in responsive communication.

Most interventions based on behavioral learning theories have been shown to improve the responsive communication of children with ASD—but not their initiating communication—through the establishment of a “stimulus–response” (Ben-Itzchak and Zachor, 2007). The ultra-light clay intervention that we developed based on applied behavior analysis and psychological development theory emphasized the understanding and internalization of communications and showed a positive effect on both the responsive and initiating communication of children with ASD. Nevertheless, the ultra-light clay intervention contains rich visual stimuli; therefore, children’s learning strategies can be continuously improved (Tissot and Evans, 2003; Hume et al., 2014). Additionally, the ultra-light clay intervention involving subject-to-subject interaction had a generalization effect on initiating communication but not on responsive communication. We speculate that the language initiated by children with ASD may include errors in pronunciation, grammar, and semantic expression (Hampton et al., 2020), making it difficult for peers to understand and respond. Future researchers can exploit some intervention methods to help children with ASD express language more accurately.

The set of ultra-light clay interventions that we designed provides some practical exercises for future interventions, including kneading exercises, making single items, creating a series of themes, creating large themes, making imaginary items, and creating imaginary themes. In addition, we provide evidence of the effectiveness of multiple forms of intervention (e.g., one-to-one, one-to-N), which may have different roles in future interventions. For example, the one-to-one form may be more effective for tracking changes in the subjects’ oral communication, while the one-to-N may be more suitable for developing the subjects’ sense of cooperation and teamwork and save the cost of interventions. However, in daily life, the social circle of children with autism includes not only special groups but also typical developmental groups. It is thus wondered whether people with autism communicate differently with individuals with autism and without. Although studies have shown that information transfer between individuals with ASD is more fluent (Crompton et al., 2020), more practical research is needed, which is a limitation of the current study. Hence, future work needs to further explore the advantages and disadvantages of these intervention forms for children with ASD. Another question worth exploring in the future is whether combining ultra-light clay intervention with behavioral reinforcement intervention will produce a more positive effect on both the responsive and initiating communication behaviors of children with ASD. We call for future studies to explore this question.

## Conclusion

Ultra-light clay intervention effectively improved the responsive and initiating communication behaviors of children with ASD and had a generalization effect between peers on initiating communication behaviors.

## Data Availability Statement

The original contributions presented in the study are included in the article/supplementary material, further inquiries can be directed to the corresponding authors.

## Ethics Statement

The studies involving human participants were reviewed and approved by Ethics Committee of East China Normal University. Written informed consent to participate in this study was provided by the participants’ legal guardian/next of kin. Written informed consent was obtained from the individual(s) and minor(s)’ legal guardian/next of kin, for the publication of any potentially identifiable images or data included in this article.

## Author Contributions

JZ, QS, and FY: generating the ideas and design. XL and QS: revising the article. JZ: refining the design and analysis of data. All authors contributed to the article and approved the submitted version.

## Funding

This present work was supported by the Zhejiang Provincial Natural Science Foundation of China (No. LY20C090011), the Humanity and Social Science foundation of the Ministry of Education (No. 18YJC630155), the Natural Science Foundation of China (No. 71801193), and Shanghai Pujiang Program (No. 13PJC037).

## Conflict of Interest

The authors declare that the research was conducted in the absence of any commercial or financial relationships that could be construed as a potential conflict of interest.

## Publisher’s Note

All claims expressed in this article are solely those of the authors and do not necessarily represent those of their affiliated organizations, or those of the publisher, the editors and the reviewers. Any product that may be evaluated in this article, or claim that may be made by its manufacturer, is not guaranteed or endorsed by the publisher.

## Footnotes

^1^In order to reduce the interference caused by Study 1, we re-selected these eight children in another school.
